# Gestational Diabetes Mellitus and Maternal Immune Dysregulation: What We Know So Far

**DOI:** 10.3390/ijms22084261

**Published:** 2021-04-20

**Authors:** Colm J. McElwain, Fergus P. McCarthy, Cathal M. McCarthy

**Affiliations:** 1 Department of Pharmacology and Therapeutics, Western Gateway Building, University College Cork, T12 XF62 Cork, Ireland; cmccarthy@ucc.ie; 2 Department of Obstetrics and Gynaecology, Cork University Maternity Hospital, T12 YE02 Cork, Ireland; fergus.mccarthy@ucc.ie

**Keywords:** gestational diabetes mellitus, immunology, inflammation, insulin resistance, mitochondrial dysfunction, therapeutics, pharmacology

## Abstract

Gestational diabetes mellitus (GDM) is an obstetric complication that affects approximately 5–10% of all pregnancies worldwide. GDM is defined as any degree of glucose intolerance with onset or first recognition during pregnancy, and is characterized by exaggerated insulin resistance, a condition which is already pronounced in healthy pregnancies. Maternal hyperglycaemia ensues, instigating a ‘glucose stress’ response and concurrent systemic inflammation. Previous findings have proposed that both placental and visceral adipose tissue play a part in instigating and mediating this low-grade inflammatory response which involves altered infiltration, differentiation and activation of maternal innate and adaptive immune cells. The resulting maternal immune dysregulation is responsible for exacerbation of the condition and a further reduction in maternal insulin sensitivity. GDM pathology results in maternal and foetal adverse outcomes such as increased susceptibility to diabetes mellitus development and foetal neurological conditions. A clearer understanding of how these pathways originate and evolve will improve therapeutic targeting. In this review, we will explore the existing findings describing maternal immunological adaption in GDM in an attempt to highlight our current understanding of GDM-mediated immune dysregulation and identify areas where further research is required.

## 1. Introduction

Pregnancy and foetal growth pose a substantial challenge for the maternal immune system. From the early stages of implantation and decidual formation, to successful foetal delivery, key maternal immunological mediators such as macrophages, natural killer (NK) cells and regulatory T cells (Tregs) must be meticulously balanced by the maternal immune system to avoid adverse pathology and/or disruption of pregnancy [1]. How various immunological mediators adapt to the state of pregnancy will thereby impact both foetal and maternal health outcomes.

Historically, an ‘allograft’ theory of early pregnancy has been presented, which suggests that maternal immunity reacts to the developing placenta and foetus as a ‘foreign’ body [2]. Genetically foreign ‘non-self’ antigens, which originate from the developing foetus, may thereby stimulate proliferation of lymphocytes such as maternal CD8^+^ cytotoxic T cells, in an antigen-specific response to pregnancy. It is then the proficiency of the maternal physiology to facilitate immunological homeostatic processes, before downstream cytolytic pathways are activated, which determines foetal survival [3]. This theory postulates that pregnancy is therefore a state of immunosuppression, leaving the mother susceptible to infection. More recent findings have, however, proposed that a maternal physiological immune profile delicately balanced between immune activation and suppression is required for a healthy pregnancy [4,5,6].

Gestational diabetes mellitus (GDM), defined as any degree of glucose intolerance with onset or first recognition during pregnancy, is a serious obstetric complication that affects approximately 5–10% of pregnancies worldwide and is characterised by an insufficient insulin response to compensate for the insulin-resistant state of pregnancy [7,8]. The pathophysiology of GDM is not fully understood, with a predominant hypothesis linking aberrant hormone expression from the placenta to maternal metabolic dysfunction and diminished insulin functionality [9]. Additional research has postulated that GDM may develop from aberrant adaption of the maternal immune system to pregnancy and upregulation of circulating inflammatory factors linked to innate immunity [10,11], instigating immune pathway dysregulation and ensuing endothelial dysfunction and vasculopathy.

Hyperglycaemia is known to cause immune dysfunction, adversely affecting neutrophil chemotaxis, macrophage function and phagocytic responses, leaving diabetic patients more susceptible to infections and related comorbidities [12]. During pregnancy, a state already defined by immunological alterations, further imbalance in innate and adaptive cellular responses will pose additional health risks, as is evident in the increased risk of hypertension and pre-eclampsia (PE), macrosomia, premature birth and stillbirth, with GDM diagnosis.

Although the significance of immune dysregulation as a causative mediator in GDM pathophysiology is currently unclear, aberrant maternal immune response contributes to secondary complications of GDM such as maternal, and foetal, cardiovascular and metabolic disorders. This stems from evidence in patients with type 2 diabetes, where immune cell infiltration of visceral adipose tissue results in the pathological disruption of insulin signalling, contributing to insulin resistance [13].

In placental tissue, histological findings from GDM placenta show evidence of villous immaturity, villous fibrinoid necrosis, chorangiosis, and increased angiogenesis with increased overall size [14]. These pathological changes were evident in 6 of 13 placental tissues from GDM pregnancies, independent of the level of glycaemic control, suggesting other sources of placental dysfunction [15]. Hyperglycaemia in GDM is associated with increased placental inflammation [16], where excessive glucose can stimulate NOD-, LRR- and pyrin domain-containing protein 3 (NLRP3) inflammasome activation in trophoblasts, inducing generation of IL-1β and IL-18 inflammatory cytokines [17]. Similarly, maternal adipose tissue dysfunction appears to be implicated in GDM pathophysiology. A strong association has been found between maternal visceral adipose tissue (VAT) mass, a key component in the generation of free fatty acids (FFAs) and glucose uptake, and GDM diagnosis when measured early in pregnancy [18,19]. As pregnancy progresses, maternal adipose stores diminish, while FFA levels rise and glucose metabolism deteriorates by 40–60% relative to pre-pregnancy levels [20]. Women with GDM are prone to dysfunctional insulin-mediated suppression of lipolysis, resulting in exaggerated FFA and glucose production, and severe insulin resistance [21].

**Figure 1:**
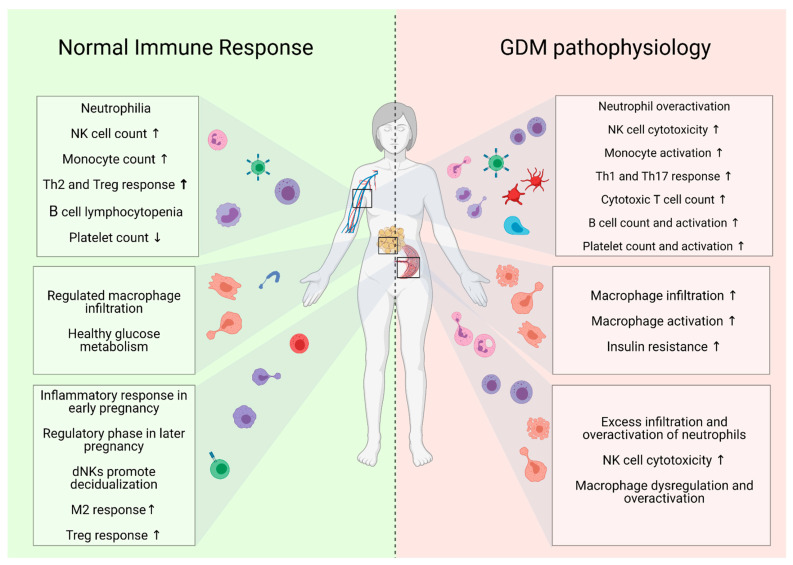
An overview characterising immune cell phenotypes in maternal circulation, adipose tissue and placental tissue in healthy uncomplicated pregnancy compared to pregnancy complicated by GDM. Created with Biorender.com. ↑, increased; ↓, decreased; GDM, gestational diabetes mellitus; NK, natural killer; Th2, T-helper 2 cell; Th1, T-helper 1 cell; Th17, T-helper 17 cell; Treg, regulatory T cell; dNK, decidual NK cell.

Considering our understanding of cellular capacity to alter tissue microenvironment and disrupt endocrine functionality, we propose the need for further research investigating the pathological contribution of circulating and tissue (namely visceral adipose tissue and placental tissue)-infiltrating immune cell populations in pregnancy (see Figure 1). In this review, we aim to explore the current landscape on how specific immune populations are altered in response to GDM pathology and examine how existing and investigational therapeutic avenues may alleviate the resultant dysregulated maternal immune response.

## 2. Immunological Regulation in Healthy Pregnancy and Its Dysregulation in GDM

### 2.1. Innate Immune Response

#### 2.1.1. Neutrophils

Neutrophils are polymorphonuclear phagocytic leukocytes that encompass the first line of immune defence against invading pathogens [22]. Initially viewed as short-lived cells with an acute role in innate immune response, recent evidence suggests that neutrophils may have long-standing effects, including CD4^+^ and CD8^+^ T cell and B cell activation, through antigen presentation in lymph nodes or neutrophil extracellular trap (NET) formation, which may play a role in pathogen eradication [23,24].

Neutrophilia and mild neutrophil activation are trademarks of healthy pregnancy, facilitated by impaired neutrophilic apoptosis [25]. Elevated maternal neutrophil counts can predict GDM pathology as early as the first trimester, where neutrophil count has been shown to be a better predictor than leukocyte count and neutrophil-to-lymphocyte ratio (NLR) for GDM diagnosis. As neutrophil count increases, GDM incidence, glycaemic levels and homeostatic model assessment of insulin resistance (HOMA-IR) all increase [26]. Other studies have also found similarly elevated neutrophil counts in GDM, proposing a significant increase in NLR in the second trimester as an assessment of inflammatory status and a robust predictor of GDM [27,28,29,30]. Serum delta neutrophil index (DNI), a relatively new biomarker of immature neutrophil count and inflammation, is also elevated when measured during the third trimester in GDM women relative to uncomplicated pregnancies [31]. Furthermore, recent evidence has reported an altered neutrophil activation profile in women with GDM versus healthy controls, where isolated neutrophils from GDM patients had increased activation and NET formation in vitro. This pro-NETotic hyperactivity was stimulated by elevated circulating levels of TNF-α in GDM plasma, suggesting that GDM pathology exhibits similar neutrophil behavioural changes as noted in other diabetic complications [32].

In the placenta, successful implantation and pregnancy require an initial inflammatory response favouring tissue remodelling, followed by a regulatory response phase to support angiogenesis and reduced allogeneic-type responses [33]. Croxatto et al. demonstrated that neutrophils may play a role in this angiogenic phase by infiltrating the decidua basalis during the first trimester. These infiltrated neutrophils were more abundant in cases of healthy pregnancy rather than spontaneous abortion. These cells tend to accumulate in close proximity to natural cytotoxic receptors^+^ group 3 innate lymphoid cells (NCR^+^ILC3), which are thought to influence neutrophil migration and survival through the production of IL-8 [33]. Primary neutrophil cultures, isolated from both healthy and GDM placental tissues, demonstrate a higher incidence of NET formation in GDM isolates relative to healthy controls. Furthermore, histological analysis confirmed the presence of excess neutrophil infiltrates in these GDM placental tissues. The pathogenic effect of hyperglycaemia on neutrophil activation in the placenta was also investigated where in vitro stimulation of neutrophils in hyperglycaemic conditions, co-cultured with BeWo trophoblasts, resulted in enhanced neutrophil activation and NET formation, which was partly mediated by trophoblast production of pro-inflammatory TNF-α, a promoter of neutrophil migration [32]. IL-8, a cytokine with specificity for neutrophil migration and activation, is also increased in GDM placenta [34]. Neutrophil overactivation may further exaggerate deleterious glucose metabolism, as exogenous neutrophil elastase liberated by degranulating neutrophils disrupts trophoblast physiology and glucose metabolism by reducing insulin receptor substrate 1 (IRS1) and glucose transporter type 4 (GLUT4) expression [35].

#### 2.1.2. NK and NKT Cells

NK cells are innate immune cells which mediate anti-tumour and anti-viral responses through strong cytolytic characteristics and comprise approximately 5–20% of human circulating lymphocytes [36]. However, recent research has proposed a novel view of NK cells as adaptive in nature, through the expression of various activating and inhibitory receptors and the development of antigen-specific immunologic memory [37].

Peripheral NK cells undergo substantial pregnancy-induced alterations. Circulating NK cell immune response appears to be amplified during pregnancy, with a greater frequency of NK cells expressing CD107a, a marker of cytolytic activity, and increased interferon (IFN)-γ expression [38]. Increased expression of CD38 and NKp46, surface markers of activation and cytotoxicity, is evident in peripheral NK cells of pregnant women versus non-pregnant women, demonstrating an immunological protective role in pregnancy.

Natural killer T (NKT) cells are a rare subset of T cells which express both an invariant αβ T cell receptor (TCR) and various surface antigens characteristic of NK cells, producing both type 1 and type 2 T helper cell-related cytokines in response to CD1d presentation from antigen-presenting cells (APCs) [39]. NKT cell function lies at the border between innate and adaptive immunity, with uncertainty regarding their role in pregnancy and the effect of increased circulating oestrogen and progesterone levels on these cell subtypes [40]. NKT cells have been shown to accumulate in decidual tissue, at ten times higher number than that observed in maternal peripheral blood, during the early stages of pregnancy. Here, these cells produce both IFN-γ and IL-4 suggesting a role in contributing to the Th1/Th2 balance at the foetal–maternal interface. However, overstimulation of decidual NKT cells appears to promote spontaneous abortion, with precise immunoregulation seemingly essential [41,42,43]. The number of peripheral NKT cells remains constant throughout pregnancy with minimal phenotypic changes. However, activation of these cells increases during the third trimester, where they produce a type 2-like cytokine response with diminished IFN-γ and increased IL-4 secretion [44].

In women with GDM, a shift towards enhanced cytotoxic capacity was recorded by elevated peripheral cytotoxic NKCD16^+^56^dim^ cell counts in overweight GDM women, compared to overweight women with uncomplicated pregnancies [45]. Circulating NKCD56^dim^ cells exhibit high cytotoxicity, whereas NKCD56^bright^ cells demonstrate low cytotoxic capacity but increased levels of cytokine release. No difference in NKCD56^bright^ cell count was noted in this study between overweight GDM women and overweight women without GDM. Chiba et al. found that GDM women had lower circulating CD56^bright^ NK cells relative to non-GDM pregnant women. Furthermore, in women with GDM, CD56^bright^ and CD56^dim^ NK cell production of IFN-γ and TNF-α was increased while there was a reduction in TGF-β expression by CD56^bright^ and CD56^dim^ NK cell populations and VEGF expression by CD56^bright^ NK cells [46]. Increased circulating CD16^+^56^−^ and reduced CD16^+^CD56^+^ NK cell counts were similarly noted by Hara et al. in GDM pregnancies [34]. These findings of a predominantly cytotoxic NK cell phenotype were also defined by gene expression profiling of Chinese GDM women, where NK cell-mediated cytotoxicity pathways were significantly associated with GDM diagnosis [47].

NKT cell subgroups demonstrate varying effects in obesity-induced inflammation, where classical invariant NKT cells appear to induce insulin resistance by inflammatory cytokine secretion, particularly in response to obesity-induced lipid excess, but the non-classical NKT subgroup promotes insulin sensitivity through the secretion Th2-type cytokines [48,49]. Although some recent clinical studies have found no significant alterations in NKT cell counts in the peripheral blood of women with GDM [50,51], further research is needed to define their potential role in in GDM.

Decidual NK (dNK) cells promote decidualization via immune tolerance and vascularisation [52]. Unlike peripheral NK cells (CD56^dim^), dNK cells (CD56^bright^) are non-cytotoxic cells with an angiogenic capacity that seem crucial for functional decidual angiogenesis [53]. While many studies suggest dNKs are derived from circulating NK cell populations, others propose that resident cells in the uterine tissue may contribute significantly to their cellular phenotype [54,55]. DNKs have shown enhanced cytolytic response to viral infections, such as human cytomegalovirus, during pregnancy, where these cells can infiltrate infected tissue areas and co-localise with infected cells, inducing dNK phenotypic changes and cytotoxic functionality [56]. Similar to the circulating immune profile in women with GDM, the placental extravillous layer of GDM and T2DM women contain an increased number of cytotoxic CD16^+^CD56^dim^ NK cells relative to mild gestational hyperglycaemia (MGH). However, whether these are resident dNK cells or cell infiltrates remains unclear [34].

#### 2.1.3. Monocytes

Monocytes, characterised by the expression of the CD14 receptor antigen, are critical defence cells in the innate immune response and comprise three subtypes: the main ‘classical monocytes’ (CD14^++^CD16^−^), which have significant phagocytic activity, and two additional subtypes, the ‘non-classical’ (CD14^+^ CD16^++^) and the ‘intermediate’ (CD14^+^ CD16^+^) [57]. Monocytes promote an innate immune response by recognising pathogenic molecules via pattern recognition receptors and respond through phagocytosis, antigen presentation, chemokine secretion and proliferation. These cells also exhibit high plasticity, enabling them to alter their phenotype according to their local microenvironment, facilitating differentiation into monocyte-derived macrophages or monocyte-derived DCs [58,59].

As maternal innate immunity adapts to the initial stages of pregnancy, the number of circulating monocytes increases significantly, particularly the nonclassical and intermediate subtypes [60]. Placental cellular products are believed to act systemically in activating monocytes, with increased expression of surface activation markers such as CD11b, CD54 and CD64. Circulating monocytes also show increased expression of IL-12 and IL-1β relative to non-pregnant women. Monocyte activation proportionally increases from the first trimester through to late gestation, confirming the critical role of innate immunity in maintaining a healthy pregnancy [61].

A recent case–control study found that the percentage of circulating CD14^+^ cells, were increased in GDM pregnancies. However, lower frequencies of intermediate monocytes (CD14^+^CD16^+^) and circulating monocytes (classical, intermediate and non-classical) expressing Toll-like receptor 4 (TLR-4) were observed in GDM, while soluble CD14^+^ serum levels (sCD14^+^) were higher in GDM patients compared to controls [62]. SCD14^+^ is a complex moiety that can stimulate either pro-inflammatory or anti-inflammatory responses and has been defined as a nonspecific marker of monocyte activation [63]. Further clinical assessment has confirmed that pregnancy-associated hyperglycaemia conditions, including GDM, have higher absolute monocyte counts with increased monocyte-lymphocyte ratios (MLR) [64]. The described increase in circulating monocyte numbers and activation levels in GDM may be linked to elevated levels of circulating monocyte chemoattractant protein-1 (MCP-1), a key factor in the recruitment and activation of both peripheral blood and adipose-resident leukocytes. Telejko et al. reported that MCP-1 significantly correlated with fasting glucose levels in GDM patients, where MCP-1 levels increased with higher plasma glucose levels in these women [65]. Additionally, significantly elevated circulating MCP-1 levels were detected in the third trimester of GDM pregnancies [66].

#### 2.1.4. Macrophages

Macrophages are APCs, derived from monocyte differentiation, with distinct phenotypic classification into either M1-like, pro-inflammatory and microbicidal, or M2-like, anti-inflammatory and immunoregulatory subtypes [67]. These subtypes are characterised by specific markers such as human leukocyte antigen–DR isotype (HLA-DR), CD11c and CD86 on M1-like macrophages and CD163, CD204, CD206 and vascular endothelial growth factor (VEGF) on M2-like macrophages [68]. During pregnancy, macrophages play an important role in mediating tolerance at the maternal-foetal interface, comprising 20–30% of decidual leukocytes [69]. M1/M2 polarization of the decidual macrophage population has been characterised by various markers such as CD11c and CD209, segregating subtypes based on lipid metabolism and inflammatory profiles [70,71,72]. Recently, a combination of CCR2 and CD11c markers have been used to characterise decidual macrophages into three distinct subtypes. CCR2^-^CD11c^low^ were the most abundant decidual macrophage population (approx. 80%), expressing mainly M2-like macrophage genes. CCR2^+^CD11c^high^ macrophages (10–15%) expressed increased levels of pro-inflammatory M1-like macrophage genes and localised with extravillous trophoblast cells (EVTs). A novel decidual macrophage population expressing CCR2-CD11c^high^ was lowly abundant (approx. 5%) and localised to proximal EVTs, while expressing M2-like macrophage genes [73].

Specific placental tissue-resident macrophages, namely Hofbauer cells (HBCs), are located in the chorionic villi and are largely associated with regulatory and anti-inflammatory functions. These cells are of foetal origin and their role in pregnancy is not yet fully understood. However, their function is believed to be altered in obstetric complications such as PE and GDM [74]. The function of HBCs resembles activated M2-like macrophage subtypes, playing an important role in placental vasculogenesis and angiogenesis. High-dimensional flow cytometry analysis identified ten functionally diverse HBC subsets present throughout pregnancy, with varying expression of activation markers such as CD14, CD68 and HLA-DR. HBCs may also produce pro-inflammatory cytokines that damage the villous cell barrier and induce localised fibrosis. Nonetheless, the described characteristics of HBCs suggest a regulatory, tissue remodelling role in the placenta rather than that of an inflammatory mediator [75].

Although, to date, no studies have explicitly measured peripheral macrophage counts in women with GDM, there has been some research into the molecular links between macrophage activity and GDM pathophysiology. Upon activation, macrophages shed their haemoglobin-haptoglobin scavenger receptor CD163 (sCD163), rendering it useful as an in vivo marker of macrophage activation [76]. Ueland et al. found that women with GDM had increased circulating concentrations of sCD163 as early as 14–16 weeks gestation and 5 years postpartum, compared with women with uncomplicated pregnancies, independent of BMI and other risk factors, proposing monocyte/macrophage activation as an important early mediator in GDM pathology [77]. These women also had reduced circulating adiponectin levels, a regulatory adipokine which protects against insulin resistance and diabetic complications. Adiponectin also regulates macrophage proliferation and function, suggesting a link between adipose tissue dysfunction and concurrent imbalance in peripheral immune regulation. Transgenic murine models expressing human adiponectin have shown that adiponectin expressed in macrophages can physiologically modulate metabolic activities such as insulin sensitivity in vivo by improving metabolism in distal tissues [78]. Similar results found that the significant increase in circulating sCD163 levels in GDM women correlated with increased sCD163 release from both placenta and adipose tissue, proposing these endocrine organs as a source of the circulating risk predictor [79]. Elevated circulating levels of macrophage migration-inhibitory factor (MIF) have also been associated with GDM [80]. MIF is a ubiquitously expressed pro-inflammatory cytokine that can stimulate Th1-type responses and IL-17 release and increases TLR-4 expression on macrophages, priming them for inflammatory stimulation [81]. Furthermore, MIF-associated genotypes are susceptibility factors in the pathogenesis of GDM [82,83]. These results further indicate a potential interplay between maternal monocytes/macrophage activation and metabolic adipokine production as a culprit in the early development of GDM.

In GDM, HBCs were shown to maintain their M2-like anti-inflammatory phenotype, characterised by CD206 and CD209 expression, with no significant difference in cytokine production relative to uncomplicated pregnancies [84]. This study was, however, limited by a small sample size. Further research found increased expression of CD163 on macrophage-like cells in GDM placental tissue samples, specifically within the chorion and decidua, with no difference in CD68 expression, suggesting a decrease in the M1/M2 ratio and a dominant anti-inflammatory phenotype [85]. Bari et al. found similar results, whereby CD163^+^ cells were significantly increased in GDM placenta compared with controls. GDM placental explants also released higher concentrations of sCD163 [79]. As described previously, this activation marker is also elevated in the circulation of women with GDM. Additional studies have observed a pro-inflammatory shift towards an M1-like phenotype, with increased CD68^+^ cells and IL-6, IL-8, TNF-α and TLR-2 expression in GDM placental tissue compared to uncomplicated pregnancies [86,87]. Gene expression of macrophage markers (CD68, CD14 and EMR-1) were also increased in GDM placenta compared with controls [88]. Similar to the circulating levels of MIF in women with GDM, placental MIF expression is increased in GDM and this correlates with maternal insulin resistance [89]. Furthermore, recent in vitro evidence has shown that palmitic acid, as a model of elevated saturated fat, induces NLRP3 inflammasome activation in primary placental macrophages isolated from GDM term placental tissue. This activation led to increased IL-1β expression and apoptosis [90]. These limited clinical findings show contrasting results in the role that macrophages and HBCs may play in GDM-related placental dysregulation.

Macrophage infiltration is significantly increased in the omental visceral adipose tissue of women with GDM compared to uncomplicated pregnancies, which significantly correlates with maternal insulin resistance [91]. Bari et al. found significantly increased CD163^+^ cell counts in subcutaneous adipose tissue in GDM pregnancies relative to uncomplicated pregnancies. These adipose tissue explants secreted higher concentrations of sCD163, a potential macrophage-derived inflammation biomarker, linking a GDM-mediated increase in adipose tissue macrophage infiltration to upregulated cellular activation and inflammation [79].

#### 2.1.5. Dendritic Cells

Dendritic cells (DCs) respond to both exogenous and endogenous antigens with resultant priming and stimulation of T cells and concurrent cytokine release [92]. Comparable to monocytes and macrophages, DCs are members of the mononuclear phagocyte system (MPS). However, their role in immune response is tailored towards antigen presentation [58]. In uncomplicated healthy pregnancy, DCs demonstrate significant phenotypic and functional heterogeneity, signifying their valuable ability to adapt to the requirement of pregnancy. DC function is altered to favour immune tolerance of foetal development and gestational maintenance, and a large part of this response relates to priming of T cell recruitment. It has been postulated that during pregnancy, circulating DCs lean towards stimulating a T-helper 2 anti-inflammatory response through lower expression of CD86 and HLA-DR and increased IL-10 release, relative to non-pregnant women [93]. Shah et al. confirmed these changes in DC function and T cell response in pregnancy, which is particularly pronounced in the later stages of pregnancy [94]. They found that circulating plasmacytoid DCs (pDCs), which are major producers of IFN, were increased relative to myeloid DCs (mDCs), which regulate pro-inflammatory responses. These alterations were closely related to T cell stimulation with a shift in T cell maturation towards an CD4^+^ effector memory subtype, particularly in the second and third trimester. Overall, it seems that DCs drive a dampened immune activation response in healthy pregnancy, and that decidual DCs which fail to recognise and adapt to implantation pose a considerable threat to foetal survival and maternal health [95].

DCs regulate the innate response in maternal-foetal interaction and impaired DC function has been reported in obstetric conditions such as IUGR and spontaneous abortion [96,97]. These cells play an integral role in response to viral pathogens, largely through the production of type I IFN, a hallmark of anti-viral immunity. DCs, particularly pDCs, detect viruses via endosomal TLR7-9 and are responsible for the first waves of IFN release in response to viral pathogens [98]. To date, only one study has quantified DC numbers in women with GDM and these results found no significant change in the peripheral DC immune profile between GDM and uncomplicated pregnancies [99]. However, this understudied cell population may play a key role in GDM pathology as recent findings suggest that obesity and hyperglycaemia alter DC function and phenotype, promoting vascular inflammation and insulin resistance [100].

#### 2.1.6. Platelets

Platelets are specialized blood cells that play central roles in physiologic and pathologic processes and maintain vascular homeostasis through mechanisms such as vasoconstrictor secretion, clot promotion and clot dissolution [101]. Platelet counts decrease during uncomplicated pregnancy, beginning in the first trimester (approx. 251,000 per cubic millimetre) and continuing to reduce until delivery (approx. 217,000 per cubic millimetre) [102]. Other than haemodilution, the reduction in platelet count may relate to accelerated sequestration and consumption in placental circulation [103]. In some cases, platelet counts can fall below healthy limits, referred to as gestational thrombocytopenia. Although thrombocytopenia has been identified as a symptom rather than a precursor to gestational complications, the incidence of complications such as PE is higher in women with previous platelet function disorders [104]. Pregnancy is itself a pro-coagulant state where platelet sensitivity to activation is upregulated by a simultaneous increase in ambient pro-thrombotic agents, thromboxane A2 (TXA2) and calcium, as well as a decrease in anti-thrombotic factors such as intraplatelet cAMP [105]. Such changes may be triggered by elevated levels of progesterone during pregnancy [106]. Therefore, platelets are ‘primed’ by various gestational adaptions to respond to stimulus. However, overactivation of platelets can stimulate damaging inflammatory pathways linked with venous thromboembolism and vascular disease, and has been linked to obstetric conditions such as PE and GDM [107,108].

Platelets also play a regulatory role in decidual tissue, where villous-adherent platelets in first trimester placental tissue were found to express inflammatory and antiangiogenic cytokines such as chemokine ligand 5 (CCL5) and chemokine ligand 4 (CXCL4), confirming their ability to regulate early placental development [109]. Similarly, platelet-derived growth factor (PDGF) has been suggested to cause proliferation of vascular smooth muscle cells in spiral arteries of the decidua, contributing to atherosclerotic changes in the pathology of pregnancy-induced hypertension [110].

Platelets are predominantly in a state of overactivation in women with GDM. Multiple studies have examined circulating platelet activity in GDM pregnancies and found that platelet count, platelet to lymphocyte ratio (PLR) and mean platelet volume (MPV), a measure of platelet function and a marker of activation potential, are significantly higher in GDM pregnancies [27,29,30,111,112,113,114,115,116,117,118,119]. A higher MPV, and larger platelet size, increases platelet aggregation and thromboxane A_2_ production, stimulating further thrombotic events and contributing to the vascular complications evident in GDM. Although some research has found no difference or diminished platelet counts and activity in GDM [114,115,120,121], the predominant hypothesis remains that GDM pathology is characterised by abnormal platelet hyperactivity and that platelet counts may present a predictive tool in GDM diagnosis.

### 2.2. Adaptive Immune Response

#### 2.2.1. T Cells

T cells are lymphocytes which play a key role in cell-mediated immunity, predominantly in the adaptive immune response, where they provide host defence against harmful microorganisms [122]. Originating in the thymus, T cells present with a specific TCR on the cell surface [123]. The presence of CD3 as a surface protein complex is a defining feature of T cell populations, where it acts as a T cell co-receptor that associates with the TCR [124]. Subsets of CD3^+^ T cells play a variety of roles in host defence mechanisms. T cell phenotypes include the classical T-helper 1 and 2 (Th1 and Th2) cells in addition to other functional subtypes including Th17 cells, Tregs and cytotoxic T (Tc) cells [122].

During the early stages of pregnancy, maternal immunity responds to the presentation of paternal-derived antigens and the associated immunological response influences both maternal and foetal health outcomes. Upon antigen presentation, effector CD4^+^ T cells release a plethora of cytokines and the profile of cytokines released from the cell population define their classification [125]. CD4^+^ T cells, also known as T-helper cells, are largely responsible for regulating effective immune responses to pathogens [126]. CD4^+^ Th1 cells predominately mediate intracellular (IC) phagocyte-mediated immune response through secretion of IL-2, tumour necrosis factor (TNF)-β and IFN-γ, whereas Th2 cells are protective against extracellular (EC) pathogens through the production of IL-4 and IL-5 [127]. An established shift from Th1 cytokine release towards Th2 cytokine release contributes to a healthy and successful pregnancy, while failure to alter this Th1/Th2 ratio has been implicated in the pathophysiology of pregnancy loss and obstetric complications [127]. Th17 cells have also been shown to contribute to a successful pregnancy, as well as being implicated in the pathophysiology of obstetric conditions, through the secretion of IL-17A, IL-17F and IL-22. These cytokines play a crucial role in endothelial defence mechanisms, particularly against fungi and bacterial pathogens, and have a widespread distribution throughout the immune system [128]. During pregnancy, most IL-17 production originates from CD4^+^ T cells in the peripheral blood and the decidua [129]. In vitro findings have suggested that IL-17 stimulation can increase the invasive capacity of the JEG-3 trophoblasts and upregulate their progesterone secretion [130,131]. Third trimester levels of circulating IL-17 were found to be increased in healthy pregnancies, suggesting a role in inflammatory modulation and possibly childbirth [132]. These results implicate Th17 cells in establishing and maintaining a healthy maternal response during pregnancy.

Tregs are another subgroup of CD4^+^ T cells and are largely responsible for moderating tolerance to self-antigens [133]. Tregs play an essential role in promoting foetal growth and survival through conditioning the maternal immune system to avoid recognising paternal semi-allogeneic tissues [134]. CD25^high^FOXP3^+^ Tregs are predominant in decidual tissues during pregnancy and help establish maternal immune tolerance to invading foetal EVTs [135]. Several studies have suggested that obstetric conditions such as PE are associated with a deficiency in Treg numbers [136,137].

In response to pathogenic invasion, CD8^+^ Tc cells are primed by APCs, instigating substantial differentiation and widespread migration within the body. This interaction allows for these cells to provide rapid and extensive immunological host defence whilst protecting against bystander tissue damage [138]. During pregnancy, these cells require careful homeostasis to continue providing host protection whilst allowing foetal cell development, without a deleterious response. Foetal-specific CD8^+^ T cells have been detected in maternal blood in the early stages and throughout pregnancy, suggesting a role for a foetal-derived immune response in regulating the maternal physiological response to pregnancy [139]. These cells were found to largely exhibit an effector memory phenotype and carried functional capacity for proliferation, cell lysis and IFN-γ secretion. A large proportion of these cells retained their functionality postnatally.

We have become accustomed to the concept that T cells subtypes play a fixed role in immune response pathways but recent evidence suggests that, under certain conditions, seemingly committed T cells demonstrate plasticity and morph into other types of effector cells, suggesting a more complex role in moderating the dynamic physiology of pregnancy [140].

In GDM, recent evidence suggests that the development and function of both naïve and memory Treg populations are altered, with reduced expression of suppressive Treg subtypes such as CD4^+^CD127^low+/-^CD25^+^ Tregs and CD45RA Tregs [141], inferring a dampening of the anti-inflammatory response of Tregs in GDM. This reduced circulating Treg count was noted as early as in the first trimester in women with GDM, where diminished number of Treg cells correlated with elevated IL-6 and TNF-α concentrations [142]. Another recent study found that GDM pregnancies have a significantly lower frequency of CD4^+^CD25^bright^ and CD4^+^CD25^+^ FOXP3^high^ cells, while TNF-α expression by circulating CD4^+^CD25^+^FOXP3^+^CD127^−^ Tregs was upregulated in these women, relative to glucose-tolerant pregnancies [45]. On the contrary, other studies have characterised third trimester GDM pregnancies with a higher ratio of activated and memory T cell phenotypes, including CD4^+^CD25^+^ Tregs, CD4^+^HLA-DR and CD4^+^CD45RO^+^ cells, with diminished naïve T cell count [50,143].

Supporting a pro-inflammatory T cell phenotype, both CD4^+^ and CD8^+^ (cytotoxic) T cell populations are elevated in the maternal circulation of women with GDM, with significantly higher treatment-independent expression of the surface activation marker CD69 and higher expression of HLA-DR in insulin-treated cases, showing exacerbated T cell activation [51]. Sifnaios et al. found evidence of low-grade inflammation in GDM patients, with increased circulating levels of high sensitivity C-reactive protein (hsCRP). However, these GDM patients had an overall higher proportion of peripheral IL-13 expressing T cells (Th2-type response), Th17 cells and IL-10 expressing Treg cells at 28–34 weeks’ gestation compared to uncomplicated pregnancies. Evidence of a pro-inflammatory T cell phenotype was confirmed in additional studies, with increased numbers of circulating Th17 cells and higher Th17:Treg and Th1:Treg pro-inflammatory cell ratios at 37 weeks gestation in women with GDM compared to women with uncomplicated pregnancies [11,144]. Additionally, gene expression of Th1 transcriptional factor (T-bet) and Th1 cytokines (IL-2 and IFN-γ) are significantly increased in obese women with GDM at term, relative to control women [145]. These findings suggest that inflammatory dysregulation in GDM may be more complex than Th1/Th2 imbalance, and that a Th1/Th2/Th17/Treg paradigm more precisely characterises the underlying immunologic profile in normal and complicated pregnancy [146]

A predominantly inflammatory and cytotoxic T cell subtype, γδ T cells, are also increased in number in the circulation of GDM pregnancies relative to uncomplicated pregnancies [99,147,148]. These ‘unconventional’ T cells are found in very small amounts in the body but have the ability to recognize a broad range of antigens without the presence of major histocompatibility complex (MHC) molecules [149]. Overall, the role of the T cell population in GDM pathology appears ambiguous with further investigation and characterisation imperative.

#### 2.2.2. B Cells

B cells are the other major cellular components of adaptive immunity. These lymphocytes are formed in the bone marrow and play a central role in disease pathogenesis, autoimmunity and alloimmunity through antigen presentation and cytokine secretion. B cell cytokine production can be dividing into “regulatory” and “effector” functions through secretion of IL-10 and TGFβ-1, or IL-2, IL-4, IL-6, IFN-γ, IL-12 and TNF-α, respectively [150,151].

Healthy uncomplicated pregnancy is generally marked by B cell lymphocytopenia. However, recent reports suggest that B cells may undergo a subtype shift in pregnancy towards a regulatory B cell (Bregs) role, providing anti-inflammatory and protective functions [152]. Prospective human and animal observational studies have characterized significantly lower peripheral B cell numbers during pregnancy, particularly during the 3rd trimester and immediately postpartum, compared to non-pregnant controls. However, these studies showed significantly higher proportions of peripheral naïve B cells and CD24^high^CD38^high^ Bregs in pregnant women and localisation of mature B cells in lymph nodes draining the uterus and the peritoneal cavity of pregnant mice [153,154]. These findings suggest a tailored B cell haematopoiesis response during pregnancy, favouring mature, Breg cells which are localised to the uterine area.

To date, only one study has examined circulating B cell populations in GDM pregnancies, which reported that B cells are increased in GDM pregnancies and are positively associated with maternal insulin resistance, expressing significantly high levels of immunoglobuin A (IgA) [155]. Elevated IgA levels have been linked to adipose tissue inflammation and altered glucose homeostasis in obesity-related insulin resistance [156]. A similar pattern has been identified in type 2 diabetes mellitus (T2DM), whereby hyperglycaemia and hyperlipidaemia in these patients correlated with an imbalanced pro-inflammatory peripheral B cell profile with an increased percentage of CD19^+^CD23^+^ B-2 cells and a decreased percentage of CD19^+^CD23− B-1 cells [157]. As the B-2 cell subtype is considered to be predominant in adaptive immunity and inflammation relative to B-1 cells, which exhibit a regulatory nature, these findings support the theory that low-grade inflammation may be a contributing factor in the aetiology of GDM-related insulin resistance.

Evidently, circulating immune factors potentially play a significant role in the aetiology of insulin resistance and poor glycaemic control in pregnancy. GDM-mediated immune dysfunction is apparent across multiple cell populations (see Table 1). The absence of a definitive immunological profile in these patients confirms the need for extended research in this field.

## 3. Therapeutic Implications

The current first-line pharmacological intervention for the treatment of GDM, when diet and lifestyle modification fail to adequately control glycaemic levels, focuses on insulin therapy. Insulin is administered subcutaneously and maintains glucose homeostasis by acting on skeletal muscle, the liver and white adipocytes to stimulate glycogen synthesis, suppress lipolysis and upregulate glucose transport [158]. Insulin is available in a range of formulations that can be tailored for individual patient needs and has the benefit of not crossing the placenta into the foetal circulation. However, insulin administration is invasive and inconvenient for the patient, with risk of injection site infection and hypoglycaemic episodes. Recent evidence has suggested that insulin may have additional effects on the immune system as a range of cells not typically involved in glucose homeostasis, including immune cells, express insulin receptors. Both in vitro and clinical research in diabetic patients have shown that insulin can mediate anti-inflammatory pathways in these cells, through the downregulation of TLR transcription on circulating mononuclear cells [159,160]. In patients with type 1 diabetes, insulin administration can suppress inflammatory markers such as CRP, IL-1β and TNF, reduce CD14 transcription and lipopolysaccharide (LPS)-stimulated apoptosis in macrophages, and attenuate neutrophil reactive oxygen species (ROS) generation [161]. Insulin also has indirect anti-inflammatory implications via the management of hyperglycaemia, thereby reducing ‘glucose toxicity’, preventing cell stress and reducing the generation of inflammatory mediators such as advanced glycation end products (AGEs) [162].

Oral anti-diabetic pharmacological therapies such metformin or sulfonylureas may also be considered for GDM treatment. The exact pharmacodynamic profile of metformin is complex and not yet fully understood. However, it is an effective insulin-sensitising agent and reduces hepatic glucose production. Metformin has minimal risk of inducing hypoglycaemic episodes and has a reduced impact on weight gain, thereby improving patient outcomes [163]. Although metformin crosses the placenta, several studies assessing foetal outcomes in metformin-treated pregnancies and have indicated that it’s safe for use in pregnancy [164,165]. Evidence suggests that metformin suppresses immune responses through induction of cellular adenosine monophosphate-activated protein kinase (AMPK) and subsequent inhibition of mechanistic target of rapamycin complex 1 (mTORC) [166]. Inhibition of mTORC results in increased IL-12 production and altered antigen presentation in DCs, and increased expression and proliferation of Tregs [167]. In high fat-fed mice models, metformin improved obesity-related inflammation by inducing M2-like macrophage polarisation, also via AMPK dependent mechanisms, resulting in decreased circulating concentrations of IL-6 and TNF-α and an overall anti-inflammatory phenotype [168]. In vitro findings show that metformin also attenuates mitochondrial ROS production, reducing mitochondrial-mediated apoptosis and oxidative stress responses [169].

Sulfonylurea drugs, such as glyburide, appear to be more effective than metformin in managing GDM-induced hyperglycaemia. However, some studies have suggested a correlation with PE, macrosomia and neonatal hypoglycaemia [170]. The predominant clinical effect of glyburide is through the stimulation of pancreatic insulin release [171]. However, glyburide also stimulates an anti-inflammatory response, particularly through M2-like polarisation and inflammasome inhibition in macrophages, which reduces the release of pro-inflammatory cytokines such as IL-1β and IL-18 [172,173]. These findings warrant further investigation into the role of treatments such as insulin, metformin and glyburide on immunoregulation in GDM pathophysiology.

The availability of therapeutic options for obstetric complications are hindered by the ethical and clinical reservations associated with the participation of pregnant women in clinical trials for novel drug treatments. One possible avenue for expanding clinical therapy options in targeting inflammation in pregnancies may be the use of monoclonal antibodies (mAbs). Anti-TNF-α antibody treatments, such as infliximab or adalimumab, have been found to attenuate aberrant macrophage activation by significantly reducing CD163 expression on circulating CD14^+^ monocytes, and normalised circulating sCD163 levels, in patients with ulcerative colitis [76]. Infliximab also significantly reduces neutrophil priming and pro-NETotic effects of GDM sera on isolated neutrophil cultures [32]. In patients with Crohn’s disease, infliximab can restore the Th1/Th2 inflammatory balance and increase circulating Treg populations [174]. However, the data behind mAb use during pregnancy is conflicting. For example, trastuzumab and rituximab are associated with poor neonatal outcomes including prolonged neutropenia [175]. Infliximab does not cross the placenta during the first trimester but undergoes efficient placental transfer during the third trimester, and has been detected in foetal serum samples, suggesting that discontinuation during the third trimester may be beneficial to minimise foetal exposure [176]. However, a review of other studies has suggested that infliximab has minimal toxicity implications in pregnancy and appears to be a safe option for mother and child [177]. A prospective cohort study has concluded that adalimumab is a safe treatment in pregnancy, where clinical benefits of treatment exceed the clinical risks in untreated pregnancies. This study found no increased risk of adverse maternal or foetal outcomes during adalimumab administration [178].

Other potential investigational avenues for attenuating immune dysregulation in GDM pregnancies also exist. MCC950, a specific inhibitor of the NLRP3 inflammasome predominantly expressed in macrophages, reduces IL-1β and IL-18 expression by blocking inflammasome assembly and caspase-1 activation [179]. In murine models of obese, hyperglycaemic pregnancies, MCC950 successfully supressed lipid-induced hyperactivation of the NLRP3 inflammasome in placental cultures [180]. MCC950 also reduced cholesterol crystal-induced IL-1β response in explants cultures of human placental tissue, demonstrating attenuation of NLRP3-mediated placental inflammation [181].

Mitochondrial dysfunction and subsequent ROS generation have been implicated in GDM pathology, whereby elevated glucose levels drive an increase in glucocorticoids (GC) and insulin-like growth factor (IGF)-1 hormones which cause mitochondrial hyperactivity and ROS generation. Uncompensated ROS generation results in an oxidative stress response and aberrant activation of innate immunity such as increased macrophage adhesion and activation [9,182]. Furthermore, circulating mitochondrial DNA (mtDNA), a marker of mitochondrial dysfunction, can behave as a damage-associated molecular pattern (DAMP) and activate an innate immune response via TLR-9 signalling [183]. We have found significantly higher levels of circulating cell-free mtDNA in GDM patients relative to age-matched controls, suggesting a pathogenic role in GDM [184]. Antioxidant therapeutics are currently being investigated in direct targeting of mitochondrial dysfunction and oxidative stress responses. MitoQ, a mitochondria-targeted synthetic antioxidant, has been found to successfully reduce trophoblast mitochondrial stress in early hypoxic rat pregnancies [185] and has been well tolerated in rat models for the treatment of cardiovascular disease [186,187]. These positive findings have been extended into clinical trials where MitoQ has been found to safely treat age-related vascular dysfunction [188]. Further research is needed to fully determine the safety profile and clinical efficacy of MitoQ in treating oxidative stress-related complications in pregnancy. L-ergothioneine is a mitochondria-targeted antioxidant that preferentially accumulated in high oxidative stress organs through the action of a specific organic cation transporter novel type 1 (OCTN1). This water-soluble amino acid has shown promising antioxidant effects during in vitro and in vivo animal studies and has demonstrated a favourable biological safety profile in in vivo pregnancy models [189,190]. Administration of L-ergothioneine in PE rat models found a significant reduction in hypertensive symptoms and kidney mitochondria-specific H_2_O_2_ generation, suggesting a promising role for L-ergothioneine in managing exaggerated oxidative stress and inflammation [191].

**Figure 2:**
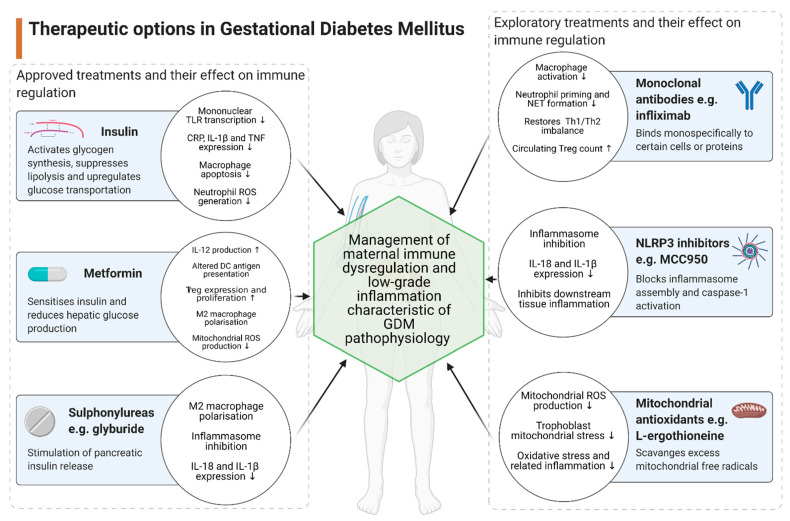
Immune-mediated effects of both clinically approved and potential therapeutic options for GDM. Created with Biorender.com. ↑, increased; ↓, decreased; TLR, Toll-like receptor; CRP, c-reactive protein; IL, interleukin; TNF, tumour necrosis factor; ROS, reactive oxygen species; DC, dendritic cell; Th1, T-helper 1 cell; Th2, T-helper 2 cell; Treg, regulatory T cell; NET, neutrophil extracellular trap; NLRP3, NOD-, LRR- and pyrin domain-containing protein 3.

Therapeutic options in GDM management are limited (see Figure 2). However, further investigation may reveal alternative avenues of pharmacological intervention.

## 4. Conclusions

Aberrant maternal immune cell adaption is key to the low-grade inflammation and poor maternal health outcomes associated with GDM diagnosis. Furthermore, GDM is associated with adverse foetal outcomes such as foetal hyperinsulinemia, macrosomia, and a greater long-term risk of diabetes mellitus and obesity [192]. Children born from hyperglycaemic pregnancies have also demonstrated altered neurological function such as deficits in attention, motor control, and perception [193]. The current understanding of this obstetric complication suggests that both innate and adaptive immune system components respond to the hyperglycaemic and insulin-resistant conditions with excessive tissue infiltration and increased cellular activation, resulting in the release of a plethora of inflammatory markers throughout the maternal system. Various metabolic organs play a role in communicating and enacting these inflammatory signals in the mother such as the placenta and visceral adipose tissue. However, uncertainty remains concerning specific immune cell populations in GDM pathology, such as how circulating levels of NKT cells and DCs are influenced, the extent of macrophage infiltration and polarisation in placental tissue and the identity and role of additional immune populations in mediating adipose tissue inflammation and metabolic dysfunction. Characterisation of infiltrating immune populations will clarify the deleterious effect that cell subgroups may have on insulin sensitivity and glucose metabolism. Therapeutic options for women with GDM are currently limited to insulin injections or a small selection of second-line oral anti-hyperglycaemic agents. Extended investigation into the complex immunological pathways involved in GDM will help to further elucidate pathophysiology and provide a window of opportunity to improve future maternal health outcomes by developing clinically effective targeted therapeutics.

## Figures and Tables

**Table 1:** A summary of the studies describing immune cell populations in maternal circulation, placental tissue and adipose tissue in GDM pregnancies and how their cellular function is altered in GDM relative to uncomplicated pregnancies. ↑, increased; ↑, decreased; ↔, no significant change observed.

Immune Cell Population		Functional Role	Studies	Implication in GDM
*In circulation*				
Neutrophils		Phagocytosis; cytokine secretion	[26,27,28,29,30,31,32]	Blood count ↑ Activation ↑
NK cells		Regulates innate immune response; cytokine secretion	[34,45,46]	NKCD56^dim^ cell count ↑ NKCD56^bright^ cell count ↓ Pro-inflammatory cytokine release ↑
NKT cells		Cytokine production	[50,51]	↔
Monocytes		Phagocytosis; antigen-presenting cells	[62,64]	CD14^+^ cell count ↑ Activation ↑ Intermediate monocyte count ↓
Dendritic cells		Antigen-presenting cells	[99]	↔
Platelets		Coagulation; vasoconstriction	[27,29,30,111,112,113,114,115,116,117,118,119,120,121]	Blood count ↑ Activation ↑
T cells	T helper cells	Inflammation; defence against IC bacterial pathogens	[11]	Th1 cell count ↑ Th17 cell count ↑
	Regulatory T cells	Regulates immune response	[11,45,141,142,143,144,145,146]	Mixed findings on Treg count and functionality
	γδ T cells	Inflammation and cytotoxicity	[99,147,148]	γδ T cell count ↑
	Cytotoxic T cells	Cytotoxicity	[51,148]	CD8+ T cell count ↑
	Naïve/memory phenotype		[50,153]	Naïve T cell count ↓ Memory T cell count ↑
B cells		Antibody secretion	[155]	Blood count ↑ Activation ↑
*Placental tissue*				
Neutrophils		Phagocytosis; cytokine secretion	[32]	Infiltration ↑ Activation ↑
Decidual NK cells		Promoting decidual vascularization	[34]	DNKCD56^dim^ cell count ↑
Macrophages		Homeostasis of placenta environment and host defence against infections	[79,84,85,86,87]	Activation ↑ Mixed findings on M1/M2 polarity
*Adipose tissue (visceral and subcutaneous)*				
Macrophages		Lipid and energy metabolism; adipocyte mitochondrial function	[79,91]	Infiltration ↑ Activation ↑

## Abbreviations

GDM	gestational diabetes mellitus
NK cell	natural killer cell
DC	dendritic cell
Treg	regulatory T cell
CTLA-4	cytotoxic T-lymphocyte-associated protein 4
IL	interleukin
TGF	transforming growth factor
cAMP	cyclic adenosine monophosphate
dNK cell	decidual NK cell
Th17	T-helper 17
PE	pre-eclampsia
NET	neutrophil extracellular trap
SGA	small for gestational age
NCR^+^ILC3	natural cytotoxic receptors^+^ group 3 innate lymphoid cells
IFN	interferon
TCR	T cell receptor
APC	antigen-presenting cell
HLA	human leukocyte antigen
VEGF	vascular endothelial growth factor
EVT	extravillous trophoblast cell
HBC	Hofbauer cell
MPS	mononuclear phagocyte system
mDC	myeloid DC
Th1	T-helper 1
Th2	T-helper 2
pDC	plasmacytoid DC
Tc	cytotoxic T cell
IC	intracellular
TNF	tumour necrosis factor
EC	extracellular
Breg	regulatory B cell
TXA2	thromboxane A2
TNF	tumour necrosis factor
EC	extracellular
Breg	regulatory B cell
TXA2	thromboxane A2
CCL5	chemokine ligand 5
CXCL4	chemokine ligand 4
PDGF	platelet-derived growth factor
HOMA-IR	homeostatic model assessment of insulin resistance
NLR	neutrophil-to-lymphocyte ratio
DNI	delta neutrophil index
TLR	Toll-like receptor
MLR	monocyte-lymphocyte ratio
MCP-1	monocyte chemoattractant protein-1
MIF	macrophage migration-inhibitory factor
CRP	C-reactive protein
T2DM	type 2 diabetes mellitus
PLR	platelet to lymphocyte ratio
MPV	mean platelet volume
NLRP3	NOD-, LRR- and pyrin domain-containing protein 3
IRS1	insulin receptor substrate 1
GLUT4	glucose transporter type 4
FFA	free fatty acid
VAT	visceral adipose tissue
SAT	subcutaneous adipose tissue
LPS	lipopolysaccharide
ROS	reactive oxygen species
AGE	advanced glycation end product
AMPK	adenosine monophosphate-activated protein kinase
mTORC	mechanistic target of rapamycin complex 1
mAb	monoclonal antibody
GC	glucocorticoid
IGF	insulin-like growth factor
DAMP	damage-associated molecular pattern
OCTN1	organic cation transporter novel type 1
